# Diffusion-weighted magnetic resonance imaging in bladder cancer: comparison of readout-segmented and single-shot EPI techniques

**DOI:** 10.1186/s40644-019-0245-1

**Published:** 2019-08-27

**Authors:** Haihu Chen, Luguang Chen, Fang Liu, Jianping Lu, Chuanliang Xu, Li Wang

**Affiliations:** 1 Department of Radiology, Changhai Hospital of Shanghai, The Second Military Medical University, No.168 Changhai Road, Shanghai, 200433 China; 2 Department of Urology, Changhai Hospital of Shanghai, The Second Military Medical University, No.168 Changhai Road, Shanghai, 200433 China

**Keywords:** Urinary bladder neoplasms, Diffusion, Echo-planar imaging, Magnetic resonance imaging

## Abstract

**Background:**

To evaluate whether readout-segment echo-planar imaging (RS-EPI) can provide better image quality in assessing bladder cancer than single-shot echo-planar imaging (SS-EPI) and to compare quantitative imaging parameters derived from both techniques.

**Methods:**

Seventy patients with bladder lesions were enrolled and underwent diffusion-weighted imaging on a 3 Tesla magnetic resonance scanner using axial RS-EPI and SS-EPI techniques. Two observers independently assessed the susceptibility, detectability, motion artefacts and blurring of the images using qualitative scores. The signal-to-noise ratio (SNR), signal intensity ratio (SIR), contrast-to-noise ratio (CNR) and ADC values of the bladder lesions were measured and compared between the two techniques and between two observers. Qualitative and quantitative comparisons of image quality were performed using the Wilcoxon signed-rank test and paired t-test. In addition, the agreement of the ADC measurements was evaluated using ICC values and Bland-Altman plots.

**Results:**

Sixty-eight patients were included in the final analysis. The scores of image susceptibility, detectability and blurring for RS-EPI were significantly higher than those for SS-EPI (all *p* < 0.05), while the motion artefact was not. There were significant differences between RS-EPI and SS-EPI in the CNR and SIR values (all *p* < 0.05) but not in the SNR or ADC values (all *p* > 0.05). The ICC values and Bland-Altman plots also showed excellent agreement between the measured ADC values of the bladder lesions.

**Conclusions:**

The RS-EPI technique provides significantly better image quality in patients with bladder cancer than the SS-EPI technique, without a significant difference in the ADC value.

## Background

Bladder cancer is one of the most common malignant tumours in urology. According to the statistics, there were 429,800 new cases of bladder cancer and 165,100 related deaths worldwide in 2012 [1]. In 2015, there were approximately 80,000 new cases of bladder cancer and 34,000 related deaths in China [2]. The treatment and prognosis of bladder cancer mainly depend on the pathological stage and grade of bladder cancer. Magnetic resonance imaging (MRI) has been used to evaluate bladder lesions, with merits such as non-ionizing radiation, good image contrast, multi-parameter imaging and high spatial resolution [3]. T1-weighted imaging has advantages in the diagnosis of fat infiltration around bladder cancer; however, the bladder wall shows hypointensity similar to that of bladder tumours on T1-weighted imaging, and the value of this method for evaluating whether the tumour has infiltrated the muscle layer is limited [4, 5]. T2-weighted imaging has good soft tissue resolution and can clearly show the relationship between bladder tumours and muscles, but it is difficult to use for distinguishing tumors from benign lesions, such as inflammation, fibrosis and oedema, leading to excessive staging [6, 7]. Dynamic contrast-enhanced MRI can be used to differentiate bladder tumours and muscles according to the different enhancement modes of the lesions, while the enhancement of small blood vessels located at the base of the tumour is similar to that of the tumour, which also leads to over-staging [8, 9]. Diffusion-weighted imaging (DWI) is a functional imaging technique that reflects the state of tissues by detecting the motion of water molecules in vivo. Bladder tumours show high signals on DWI, while benign changes, such as inflammation, fibrosis and oedema, caused by tumour growth show low signals, and the bladder wall shows slightly higher signals; therefore, DWI can be used to avoid excessive staging caused by the inability to distinguish inflammatory lesions, fibrotic changes, oedema and small vessels [6, 10, 11]. In recent years, several studies have reported that DWI plays a significant role in preoperative staging and grading for the diagnosis of bladder cancer [6, 11–14].

Single-shot echo-planar imaging (SS-EPI) is a commonly used method due to its relative insensitivity to motion-induced phase errors and short acquisition time [15]. However, SS-EPI images are characterized by blurring along the phase encoding direction due to T2* decay and are sensitive to off-resonance effects [16]. Susceptibility variation at tissue-air interfaces can result in magnetic susceptibility artefacts, especially at higher field strengths. In addition, it is usually difficult to use diffusion-weighted images with low spatial resolution that are acquired using the SS-EPI technique to assess small lesions. In recent years, a new multi-shot technique, referred to as readout-segmented echo-planar imaging (RS-EPI), has been proposed for improved the spatial resolution and decreased susceptibility-induced image distortion and T2* blurring [16]. This new multi-shot EPI technique is based on the segmentation of k-space into several partitions in the readout-encoding direction at each excitation, which allows for a reduction in the echo spacing and shortens the time required for the k-space trajectory in the phase-encoding direction. In addition, a two-dimensional navigator echo was integrated into the RS-EPI technique and used for phase correction between different shots, making the method more robust with respect to motion-induced phase errors. A number of reports have shown improved image quality using the RS-EPI technique in various tissues in vivo, such as the paediatric brain, paranasal sinus, orbit, cholesteatoma, liver, breast, kidney, prostate and rectum [17–25]. However, few studies have reported the assessment of bladder lesions using RS-EPI. We hypothesized that the RS-EPI could be a promising method for DWI in bladder cancer.

Therefore, the aims of the present study were to evaluate whether RS-EPI can provide better image quality in assessing bladder cancer than SS-EPI and to compare quantitative imaging parameters derived from RS-EPI and SS-EPI.

## Methods

### Patients

Between June 2017 and December 2017, 70 consecutively patients suspected of bladder cancer were recruited in this retrospective study. The inclusion criteria were as follows: (1) bladder cancer confirmed by preoperative cystoscopy; (2) surgical treatment performed within 2 weeks after MRI examination; and (3) postoperative pathological staging. Patients with no histological evidence of bladder cancer and magnetic resonance imaging contraindications were excluded from this study. This study was approved by the local institutional review board, and written informed consent was waived for each patient in this retrospective study.

### Magnetic resonance imaging

All patients were examined using on a 3 Tesla MRI scanner (Skyra, Siemens medical solution, Germany) with a standard 18-channel phased-array body coil and an integrated spine coil. Each patient fasted for 4 h and was instructed to drink 500 ml of water to dilate the bladder before the MRI examination. In addition, patients were given regular breathing training to reduce respiratory artefacts. The imaging protocols included axial T1-weighted turbo spin echo (TSE), T2-weighted TSE, axial DWI with RS-EPI and SS-EPI and contrast-enhanced three-dimensional T1-weighted volumetric interpolated breath-hold examination (3D T1W VIBE). The main parameters of these protocols are presented in Table 1.

**Table 1:** The main parameters of MRI protocols

Protocols	TR/TE (ms)	FOV (mm^2^)	Matrix	Thickness (mm)	Gap (mm)	Slices	Acceleration factor	b-values (s/mm^2^)	Averages	Readout segments	Scan time
T1W TSE	680/20	220 × 220	180× 256	4	0.8	30	3	–	2	–	2′2”
T2W TSE	7500/101	200 × 200	320 × 320	4	0.4	20	2	–	2	–	3′9”
SS-EPI	3000/66	176 × 220	72 × 90	4	0	16	2	0, 1000	1, 12	1	2’
RS-EPI	3400/63	156 × 260	102 × 170	4	0	16	2	0, 1000	1, 4	5	4′35”
T1W VIBE	3.33/1.23	234 × 360	154× 192	3	0	30	4	–	1	–	16”

*Note*: *FOV* field of view, *RS-EPI* Readout segmented echo-planar imaging, *SS-EPI* Single-shot echo-planar imaging, *TR/TE* repetition time/echo time, *T1W TSE* T1-weighted turbo spin echo, *T1W VIBE* T1-weighted volumetric interpolated breath-hold examination, *T2W TSE* T2-weighted turbo spin echo

### Image analysis

All images were sent to an advanced workstation (Leonardo, Siemens medical solution, Germany) for further analysis. Apparent diffusion coefficient (ADC) maps of the RS-EPI and SS-EPI images were reconstructed online in the scanner using the monoexponential mode. The RS-EPI and SS-EPI DWI images were evaluated by two independent observers (H.C and F.L., with 4 and 5 years of experience in pelvic MRI, respectively) in terms of the susceptibility, detectability, motion artefact and blurring of the lesion images using a quantitative 4-point scale (Table 2).

**Table 2:** Criteria for qualitative comparison of image quality in diffusion-weighted imaging in patients with bladder cancer

Susceptibility	
1: marked distortion was seen	
2: moderated distortion was seen	
3: little distortion was seen	
4: no distortion was seen	
Detectability	
1: not visible	
2: partial visibility	
3: visibility	
4: excellent visibility	
Motion artifact	
1: marked artifact	
2: moderate artifact	
3: slight artifact	
4: no artifact	
Image blurring	
1: the bladder wall definition was not visible	
2: the bladder wall definition was partially visible	
3: the bladder wall definition was visible	
4: the bladder wall definition was clearly visible	

**Fig. 1:**
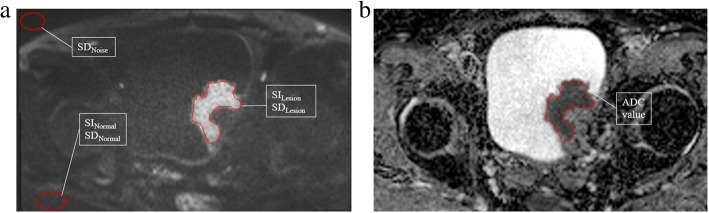
Representative images of outlined ROIs in bladder lesions. **a** DWI: b-value = 1000 s/mm^2^. ROIs were delineated to determine the signal intensity and standard deviation of the lesion and normal reference tissues, as was the standard deviation of the noise level. **b** ROI outlined for estimating the ADC value of the bladder lesion

Freehand regions of interest (ROIs) were manually outlined to profile the lesion on the ADC maps of the RS-EPI and SS-EPI images and then copied them to the corresponding diffusion-weighted images, with b-value = 1000 s/mm^2^. ROIs were drawn on the diffusion-weighted images with reference to non-contrast and contrast-enhanced T1W images to avoid the inclusion of hemorrhagic and necrotic tissues. ROIs of normal tissue were drawn in the gluteus maximus. The mean value and standard deviation of each ROI were recorded (Fig. 1). The signal-to-noise ratio (SNR) was calculated as the ratio between the mean signal amplitude of the lesion (SI_lesion_) in the diffusion-weighted images and the standard deviation of the background noise (σ_noise_). The signal intensity ratio (SIR) was defined as the ratio of the SI_lesion_ to the mean signal intensity of normal tissue (SI_normal_). The contrast-to-noise ratio (CNR) was determined as the difference between the SI_lesion_ and SI_normal_ divided by the standard deviation of the lesion (σ_lesion_) and normal tissue (σ_normal_). The SNR, SIR and CNR were calculated using the following formulas:

$$ \mathrm{SNR}=\raisebox{1ex}{${SI}_{lesion}$}\!\left/ \!\raisebox{-1ex}{${\sigma}_{noise}$}\right. $$

$$ \mathrm{SIR}=\raisebox{1ex}{${SI}_{lesion}$}\!\left/ \!\raisebox{-1ex}{${SI}_{normal}$}\right. $$

$$ \mathrm{CNR}=\raisebox{1ex}{$\left|{SI}_{lesion}-{SI}_{normal}\right|$}\!\left/ \!\raisebox{-1ex}{$\sqrt{{\sigma_{lesion}}^2+{\sigma_{normal}}^2}$}\right. $$

### Statistical analysis

Statistical analyses were carried out using SPSS (version 16.0, SPSS Inc., Chicago, IL, USA) and MedCalc (version 13.0.0.0, MedCalc Software, Mariakerke, Belgium) software. The Kolmogorov-Smirnov test was used to assess the normality of the distribution of continuous variables. Quantitative data are described as the mean ± standard deviation. Differences in image quality between RS-EPI and SS-EPI were assessed using the Wilcoxon signed-rank test. Significant differences between the two techniques and between the two observers in the quantitative parameters (SNR, SIR and CNR) of image quality and the ADC values were assessed by using paired t-test. Agreement between the two techniques and between the two observers for the ADC values were evaluated using the intraclass correlation coefficient (ICC) and Bland-Altman plots [26, 27]. ICC values < 0.4 indicated poor agreement; 0.4–0.75, good agreement; and > 0.75, excellent agreement. In addition, the mean percent difference and 95% limits of agreement between the paired ADC measurements were calculated. A *p*-value less than 0.05 was regarded as statistically significant.

## Results

### Patients

A total of 70 patients were recruited for RS-EPI and SS-EPI DWI. However, 1 patient was excluded because of the lack of histological evidence of bladder cancer, and 1 patient was excluded due to contraindications to magnetic resonance imaging. Therefore, 68 patients (60 males and 8 females, mean age: 66.1 ± 14.7 years) completed all MRI examinations. Histology showed that 49 patients had high-grade urothelial carcinoma and 19 patients had low-grade urothelial carcinoma. Pathological staging showed that 48 patients had T1, 2 patients had T2, 5 patients had T3a, 4 patients had T3b, 3 patients had T4, in addition, 6 patients were identified as having muscle invasive bladder cancer (> T1), but the exact stages were not determined because the whole bladder was not resected and pathological sections of the bladder tumour and the bladder wall could not be obtained. The tumour size ranged from 5 mm to 65 mm (mean: 26.6 ± 13.2 mm).

### Qualitative comparison of image quality

**Fig. 2:**
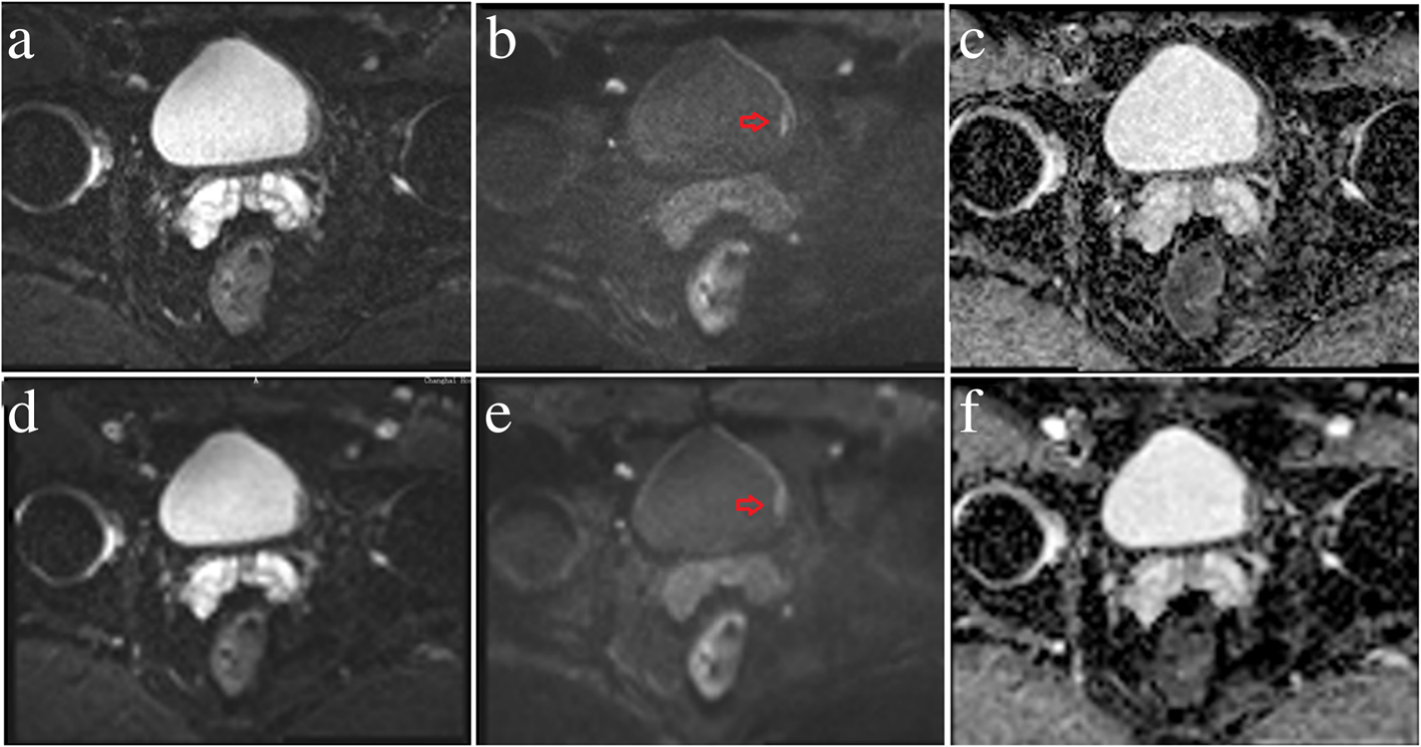
A 39-year-old male with low-grade urothelial carcinoma in the left wall of the bladder (red arrows). RS-EPI: b-value = 0 s/mm^2^ (**a**); b-value = 1000 s/mm^2^ (**b**); ADC map (**c**). SS-EPI: b-value = 0 s/mm^2^ (**d**); b-value =1000 s/mm^2^ (**e**); ADC map (**f**)

**Fig. 3:**
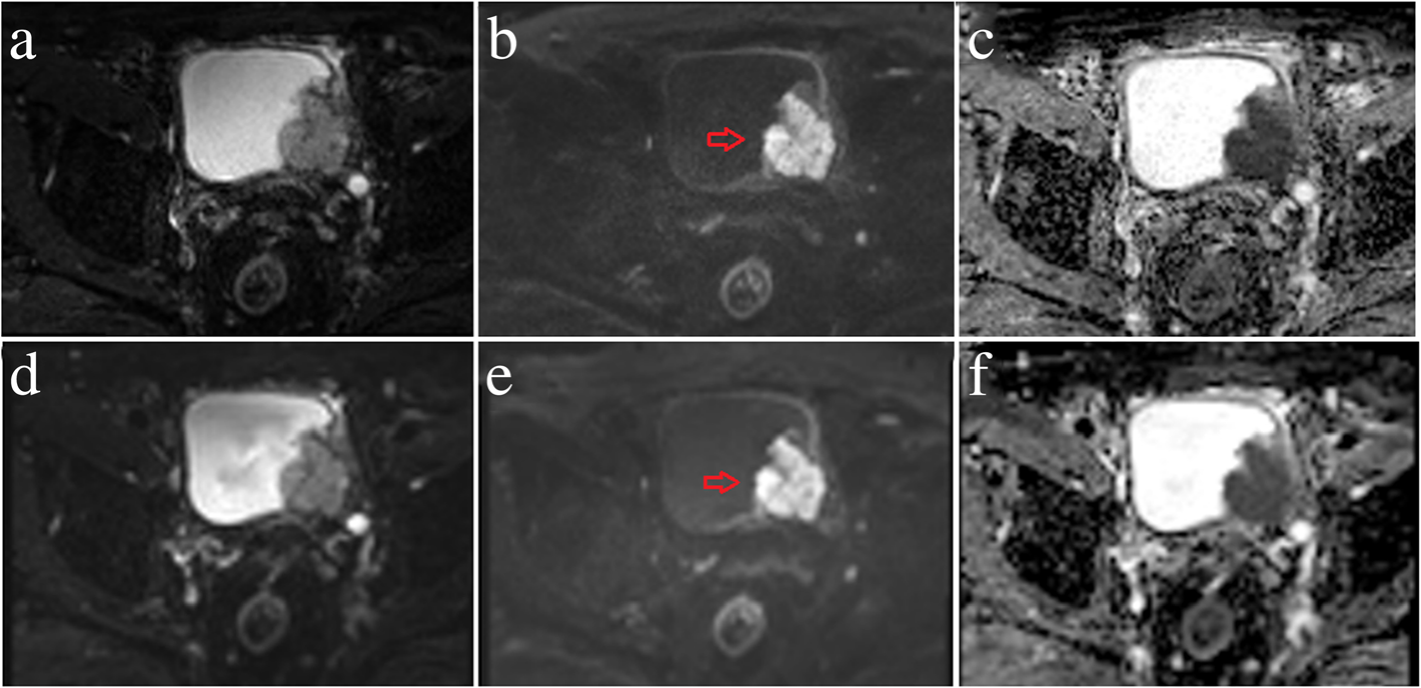
A 70-year-old male with high-grade urothelial carcinoma in the left posterior wall of the bladder (red arrows). RS-EPI: b-value = 0 s/mm^2^ (**a**); b-value =1000 s/mm^2^ (**b**); ADC map (**c**). SS-EPI: b-value = 0 s/mm^2^ (**d**); b-value =1000 s/mm^2^ (**e**); ADC map (**f**)

**Fig. 4:**
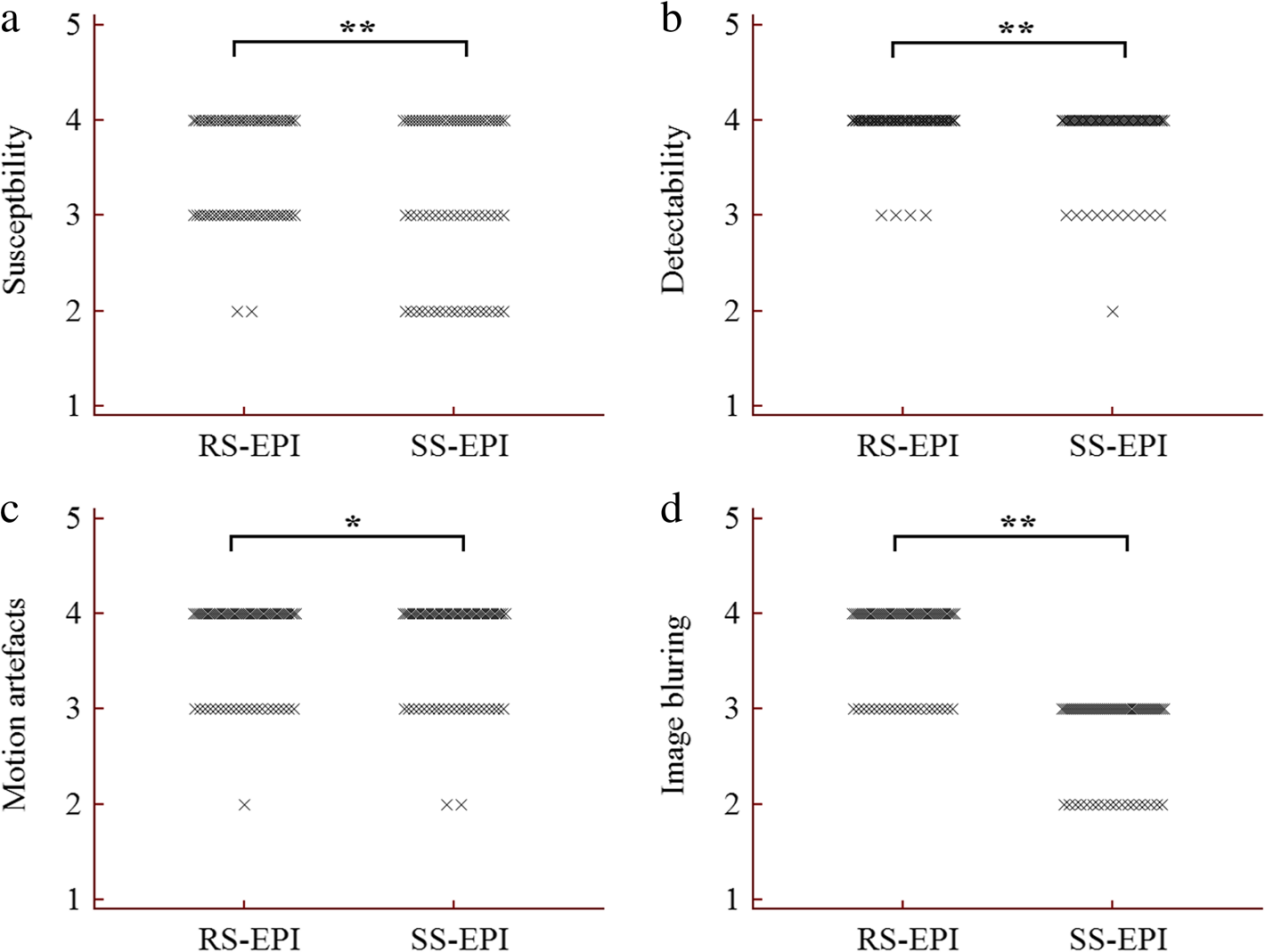
Qualitative comparison of image scores between RS-EPI and SS-EPI in patients with bladder cancer. **a** Scores for susceptibility; **b** Scores for detectability; **c** Scores for motion artefact; **d** Scores for image blurring

The results of the image quality analysis for the RS-EPI and SS-EPI techniques are shown in Table 3. There were significant differences between the two techniques in the susceptibility, detectability and blurring of the images of the bladder lesions (all *p* < 0.05) but not the motion artefacts (3.68 ± 0.50 vs. 3.63 ± 0.54 and 3.69 ± 0.47 vs. 3.66 ± 0.54, *p* = 0.083 and 0.317) for both observers. Representative images from patients with low- and high-grade urothelial carcinoma are shown in Figs. 2 and 3. Figure 4 shows dot plots of image quality for the RS-EPI and SS-EPI techniques. The mean image quality scores were higher for RS-EPI than SS-EPI.

**Table 3:** Qualitative comparison of image scores between RS-EPI and SS-EPI techniques in patients with bladder cancer

Observer	Protocol	Susceptibility	Detectability	Motion artifact	Image blurring
Observer 1	RS-EPI	3.50 ± 0.56	3.94 ± 0.24	3.68 ± 0.50	3.71 ± 0.46
	SS-EPI	3.22 ± 0.84	3.82 ± 0.42	3.63 ± 0.54	2.74 ± 0.44
	*p-value*	0.000	0.005	0.083	0.000
Observer 2	RS-EPI	3.41 ± 0.67	3.94 ± 0.24	3.69 ± 0.47	3.76 ± 0.43
	SS-EPI	3.22 ± 0.81	3.82 ± 0.42	3.66 ± 0.54	3.01 ± 0.47
	*p-value*	0.000	0.011	0.317	0.000

*Note*: *RS-EPI* Readout segmented echo-planar imaging, *SS-EPI* Single-shot echo-planar imaging

### Quantitative comparison of image quality parameters

A quantitative comparison of image quality parameters between RS-EPI and SS-EPI for the two observers is shown in Table 4. There were significant differences between the RS-EPI and SS-EPI techniques in the CNR and SIR values (all *p* < 0.05), while no significant difference was observed in the SNR value (*p* > 0.05). Table 5 shows comparisons of the SNR, SIR and CNR measurements between Observer 1 and Observer 2 for both techniques; no significant differences were observed in those measurements (all *p* > 0.05).

**Table 4:** Quantitative comparison of image quality between RS-EPI and SS-EPI techniques in patients with bladder cancer

	Observer 1			Observer 2		
	RS-EPI	SS-EPI	*p-value*	RS-EPI	SS-EPI	*p-value*
SNR	118.1 ± 61.56	122.64 ± 54.97	0.2480	125.93 ± 49.69	128.88 ± 57.28	0.6520
CNR	4.95 ± 1.39	4.18 ± 1.26	< 0.001	4.81 ± 1.36	3.99 ± 1.28	< 0.001
SIR	5.42 ± 1.45	5.18 ± 1.37	0.0230	5.57 ± 1.57	5.27 ± 1.45	0.0240

*Note*: *SNR* Signal-to-noise ratio, *CNR* Contrast-to-noise ratio, *SIR* Signal intensity ratio

**Table 5:** Quantitative comparison of image quality between the two observers in patients with bladder cancer

	RS-EPI			SS-EPI		
	Observer 1	Observer 2	*p-value*	Observer 1	Observer 2	*p-value*
SNR	118.1 ± 61.56	125.93 ± 49.69	0.150	122.64 ± 54.97	128.88 ± 57.28	0.310
CNR	4.95 ± 1.39	4.81 ± 1.36	0.236	4.18 ± 1.26	3.99 ± 1.28	0.080
SIR	5.42 ± 1.45	5.57 ± 1.57	0.083	5.18 ± 1.37	5.27 ± 1.45	0.368

*Note*: *SNR* Signal-to-noise ratio, *CNR* Contrast-to-noise ratio, *SIR* Signal intensity ratio

### Quantitative comparison of ADC values

**Fig. 5:**
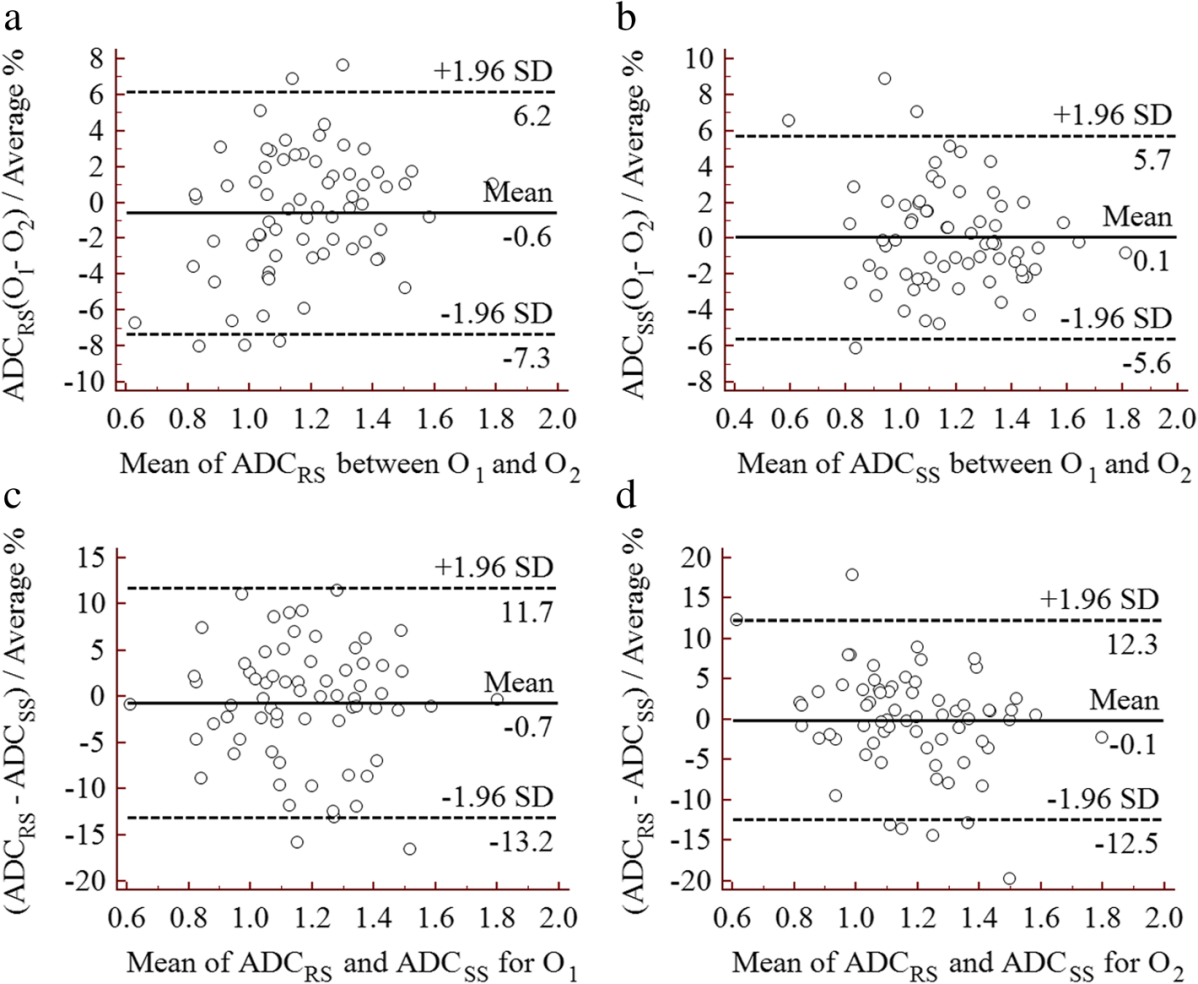
Bland-Altman plots of the ADC values for the RS-EPI and SS-EPI techniques and observers 1 and 2. **a** Comparison of ADC values derived from RS-EPI (ADC_RS_) between observer 1 (O_1_) and observer 2 (O_2_). **b** Comparison of ADC values derived from SS-EPI (ADC_SS_) between O_1_ and O_2_. **c** Comparison of ADC values measured by O_1_ between RS-EPI and SS-EPI. **d** Comparison of ADC values measured by O_2_, between RS-EPI and SS-EPI

A quantitative comparison and the agreement of the ADC values are shown in Table 6. There were no significant differences between the two techniques (1.170 ± 0.215 vs. 1.179 ± 0.221 × 10^− 3^ mm^2^/s, *p* = 0.322 for Observer 1, and 1.174 ± 0.207 vs. 1.179 ± 0.226 × 10^− 3^ mm^2^/s, *p* = 0.604 for Observer 2, respectively) for either observer, in addition, there were no significant differences between the two observers (1.170 ± 0.215 vs. 1.174 ± 0.207 × 10^− 3^ mm^2^/s, *p* = 0.302 for RS-EPI, and 1.179 ± 0.221 vs. 1.179 ± 0.226 × 10^− 3^ mm^2^/s, *p* = 0.956 for SS-EPI, respectively) obtained using RS-EPI and SS-EPI. In addition, the agreement of the ADC values between the two technique and between the two observers was excellent, with ICC values ranging from 0.937 to 0.990. Moreover, the mean percent difference and limits of agreement between Observer 1 and Observer 2 (O_1_ and O_2_) for the ADC values obtained using RS-EPI (ADC_RS_) and SS-EPI (ADC_SS_) were − 0.6 (− 7.3–6.2) and 0.1 (− 5.6–5.7), respectively (Fig. 5a, b). The mean percent difference and limits of agreement between the two techniques for the ADC values measured by O_1_ and O_2_ were − 0.7 (− 13.2–11.7) and − 0.1 (− 12.5–12.3), respectively (Fig. 5c, d).

**Table 6:** Quantitative comparison and agreement of ADC values in patients with bladder cancer

	RS-EPI	SS-EPI	*p-value*	ICC	95% CI
Observer 1	1.170 ± 0.215^a^	1.179 ± 0.221^a^	0.322	0.937	0.900–0.961
Observer 2	1.174 ± 0.207^a^	1.179 ± 0.226^a^	0.604	0.940	0.904–0.962
*p-value*	0.302	0.956	/	/	/
ICC	0.984	0.990	/	/	/
95%CI	0.974–0.990	0.984–0.994	/	/	/

*Note*: *ICC* intraclass coefficient, *CI* Confidence interval

^a^, ADC values, ×10^−3^ mm^2^/s

## Discussion

In the present study, we demonstrated significantly higher image quality and better image contrast in bladder DWI using RS-EPI than SS-EPI. In addition, no significant difference was observed in the SNR or ADC value, which shows that RS-EPI is a promising method for reducing image artefacts and improving image quality in bladder cancer. The blurring and detectability of bladder cancer lesion was significantly improved with RS-EPI. The RS-EPI technique provides significantly better image quality than SS-EPI at 3 Tesla and can potentially provide better image quality in patients with bladder cancer.

DWI is a useful MRI technique and has been used to characterize the pathophysiology of lesions in clinical practice; it offers functional and structural information on in vivo tissues without ionizing radiation or contrast administration [28]. Several studies have reported that DWI plays a significant role in routine MRI examinations and serves as a good supplement to improve the accuracy of assessing the properties and biological behaviours of bladder cancer [6, 29–31]. In addition, ADC values, which are derived from diffusion-weighted images, have been shown to have potential as imaging biomarkers for predicting the histopathological grade and indicating the aggressiveness of bladder cancer [11, 12, 14, 32, 33]. However, routine DWI is based on the single-shot EPI technique, which is sensitive to susceptibility and distortion. In the present study, a new multi-shot EPI technique (i.e., RS-EPI) was used and qualitatively and quantitatively compared in terms of image quality with SS-EPI. The qualitative assessment showed that RS-EPI is superior to SS-EPI in susceptibility, image blurring and lesion detectability. This difference may be explained by the fact that the segmented trajectory through k-space along the readout-encoding direction decreased the echo spacing and accelerated traversal of the k-space along the phase-encoding and readout directions [16]. Our findings are similar to previously reported results [18, 19, 22, 34–36]. Motion artefacts in RS-EPI images were not significantly different from those in SS-EPI images. On the one hand, while SS-EPI is insensitive to motion artefacts, a two-dimensional navigator echo was integrated into the RS-EPI technique and used to correct data corrupted because of shot-to-shot phase errors in DWI, rendering RS-EPI immune to those artefacts [16]. On the other hand, the bladder is located in the pelvic cavity and is less affected by respiratory motion artefacts. Therefore, improved image quality was obtained with the RS-EPI technique.

The CNR of bladder lesions in RS-EPI images was significantly better than that lesions in SS-EPI images, and our results are consistent with those of previous studies [20, 22, 25, 37]. Better visualization of bladder lesions can be achieved using the RS-EPI technique. However, several studies have reported no significant difference in the CNR between the two techniques [17, 18, 36]. There was a significant difference in the SIR of bladder lesions between RS-EPI and SS-EPI in the present study. The gluteus maximus was used as a reference for calculating the normalized signal intensity of the bladder lesions so that the SIR values could be compared among the patients.

The SNR of bladder lesions in RS-EPI images was similar to those of lesions in SS-EPI images, and no significant difference was observed between the techniques in this study. Our results are consistent with those of Byeon et al., who compared RS-EPI and SS-EPI in the diagnosis of acute infarction of the brain stem and posterior fossa [18]. Several studies found that the SNR of RS-EPI images was higher than that of SS-EPI images [20, 22, 24, 25, 35], while other reports showed lower SNR values for RS-EPI images [17, 19, 36–39]. The inconsistent results of those studies could be explained by the fact that the SNR is a relative value and depends on the exact imaging parameters being used, such as the magnetic field strength, minimum TE, FOV, matrix, signal average, number of segments, echo spacing, gradient performance, combination of b-values and T2 value for the tissue. In the present study, the minimum TE, in-plane resolution, number of segments and signal average were 63 ms, 1.53 × 1.53 mm^2^, 5 and 4 for RS-EPI and 66 ms, 2.44 × 2.44 mm^2^, 1 and 12 for SS-EPI, respectively, whereas the other parameters were kept consistent. Therefore, while a similar SNR was obtained using both techniques, the RS-EPI technique provided high-resolution diffusion-weighted images that contributed to the detection of small lesions (Fig. 2).

The mean ADC values of bladder lesions obtained using RS-EPI and SS-EPI were not significantly different and are in good agreement with previously reported data [17, 18, 20, 25, 35, 40]. Nevertheless, Zhao et al. found that the ADC values of sinonasal lesions on RS-EPI were lower than those on SS-EPI [36]. Moreover, Xu et al. reported that RS-EPI DWI produced significantly lower ADC values than did SS-EPI DWI in patients with orbital tumours [37]. The disagreement between the two methods in the ADC may be explained by the fact that different imaging parameters, such as the magnetic field strength, b-value, TE, spatial resolution, diffusion direction and imaging technology vendor, were used in those studies. In this study, there was excellent reproducibility between the two methods and between the two observers in the measured ADC values of the bladder lesions, as indicated by the ICC values. Moreover, the Bland-Altman plots show the excellent reproducibility of those measurements and narrow intervals of agreement compared with the average values obtained in evaluating the ADC values of bladder lesions (Fig. 5). The improved image quality of RS-EPI and equivalent ADC values derived using RS-EPI and SS-EPI suggest that we may adopt the new method as a substitute for the conventional EPI technique for imaging bladder lesions.

The present study has several limitations. First, compared with SS-EPI, the scan time for imaging bladder lesions using RS-EPI was relatively long (RS-EPI vs SS-EPI: 4 min 35 s vs. 2 min), and new techniques for accelerating the scan (e.g., simultaneous multi-slice imaging) and optimizing the imaging parameters would overcome this concern. Second, no comparison of the diagnostic efficiency of SS-EPI and RS-EPI in differentiating between high- and low-grade urothelial carcinoma was performed, and because of the relatively small sample size of patients with low-grade urothelial carcinoma, we will recruit more patients for evaluation in future studies. Third, the delineation of ROIs in the bladder lesions was based on a single-slice method in which the maximum lesion size was outlined. However, the effects of ROI methods were not compared in this study, but we will perform such comparisons in future work.

## Conclusions

In conclusion, the RS-EPI technique provides significantly improved image quality in patients with bladder cancer compared with the SS-EPI technique, without a significant difference in the ADC value. The RS-EPI DWI technique may serve as a promising tool for evaluating patients with bladder lesions in clinical practice.

## Data Availability

The datasets used and/or analyzed during the current study are available from the corresponding author on reasonable request.

## Abbreviations

ADC: Apparent diffusion coefficient
CNR: Contrast-to-noise ratio
DWI: Diffusion-weighted imaging
FOV: Field of view
ICC: Intraclass correlation coefficient
MRI: Magnetic resonance imaging
ROI: Region of interest
RS-EPI: Readout-segmented echo-planar imaging
SIR: Signal intensity ratio
SNR: Signal-to-noise ratio
SS-EPI: Single-shot echo-planar imaging
TA: Acquisition time
TR/TE: Repetition time/echo time
TSE: Turbo spin echo
VIBE: Volumetric interpolated breath-hold examination
